# LPLUNC1 stabilises PHB1 by counteracting TRIM21-mediated ubiquitination to inhibit NF-κB activity in nasopharyngeal carcinoma

**DOI:** 10.1038/s41388-019-0778-6

**Published:** 2019-03-18

**Authors:** Heran Wang, Yujuan Zhou, Linda Oyang, Yaqian Han, Longzheng Xia, Jingguan Lin, Yanyan Tang, Min Su, Shiming Tan, Yutong Tian, Xiaoyan Chen, Xia Luo, Jiaxin Liang, Shan Rao, Ying Wang, Wei Xiong, Zhaoyang Zeng, Hui Wang, Guiyuan Li, Qianjin Liao

**Affiliations:** 1 Hunan Key Laboratory of Translational Radiation Oncology, Hunan Cancer Hospital and The Affiliated Cancer Hospital of Xiangya School of Medicine, Central South University, 283 Tongzipo Road, Changsha, 410013 Hunan China; 2 Key Laboratory of Carcinogenesis and Cancer Invasion of Ministry of Education, Cancer Research Institute, Central South University, Changsha, 410078 China

**Keywords:** Cancer, Cell biology

## Abstract

Long-palate, lung and nasal epithelium clone 1 (LPLUNC1) is a tumour suppressor gene in nasopharyngeal carcinoma (NPC), and low expression of LPLUNC1 is associated with poor prognosis. Our previous study showed that LPLUNC1 upregulates Prohibitin 1 (PHB1), a pleiotropic protein that functions as a tumour suppressor gene in various cancers. Low expression of PHB1 was also found to be associated with the poor prognosis of NPC patients. However, the mechanisms by which LPLUNC1 upregulates PHB1 and the potential role of PHB1 in NPC are unclear. Here, we found that LPLUNC1 stabilised PHB1 by inhibiting PHB1 ubiquitination, which is mediated by E3 ligase TRIM21. LPLUNC1 competitively impaired the binding of PHB1 to TRIM21 due to its stronger binding affinity to PHB1, suppressing the ubiquitination of PHB1. Therefore, our study indicates that PHB1 acted as a tumour suppressor gene by inhibiting NF-κB activity. Depletion of PHB1 significantly attenuated the anti-tumour effects of LPLUNC1 in NPC cells, and the inhibitory effect of LPLUNC1 on NF-κB activity was thus reversed. Together, our findings revealed a novel mechanism underlying the anticancer effect of LPLUNC1 and clarified that PHB1 may represent a novel, promising candidate tumour suppressor gene in NPC, with potential therapeutic target value.

## Introduction

Nasopharyngeal carcinoma (NPC) is a type of malignancy with obvious regional differences that is prevalent in Southeast Asia and southern China. The initiation and progression of NPC is related to EB virus infection, environmental and dietary factors, genetic susceptibility and chronic inflammation [1, 2]. NPC is also considered an inflammation-related cancer [3, 4], but the molecular mechanisms remain unclear.

Long palate, lung and nasal epithelium clone 1 (LPLUNC1), also named BPI fold containing family B, member 1 (BPIFB1), belongs to PLUNC family. LPLUNC1 gene is a new member in BPI/PLUNC super family that encodes a protein with 484 amino acids and 2 BPI structural domains. A high level of LPLUNC1 expression can be observed in nasal pharyngeal and respiratory secretions, lavage and sputum secretions, and bronchial epithelial cell culture medium [5, 6]. Therefore, LPLUNC1 is also known as a natural immune molecule of the nasopharyngeal respiratory epithelium, regulating cellular immune response for anti-inflammation [7, 8]. LPLUNC1 is downregulated in NPC tissues and acts as a potential tumour suppressor [9, 10]. Over-expressed LPLUNC1 can repress the proliferation of NPC cells by suppressing the STAT3 signalling pathway induced by IL-6 [10]. In addition, LPLUNC1 can inhibit the migration and invasion of NPC cells and inhibits radioresistance in nasopharyngeal carcinoma by inhibiting VTN expression [11, 12]. Therefore, LPLUNC1 plays an important role in the initiation and progression of NPC. However, the molecular mechanism of LPLUNC1 in NPC development is still unclear.

To further investigate the roles and mechanisms of LPLUNC1 in NPC, we analysed the proteomic profiles in NPC cells with LPLUNC1 over-expression, and Prohibitin 1 (PHB1) was found to be upregulated by LPLUNC1 [13]. PHB1 is a mitochondrial chaperone involved in mitochondrial function and biogenesis [14, 15]. PHB1 is ubiquitously expressed in different cellular compartments and performs various biological functions, such as cell cycle, cell growth and differentiation, apoptosis and ageing [16, 17]. Recently, increasing evidence has shown that PHB1 is a tumour suppressor protein and a potential target for cancer therapy [18, 19]. PHB1 can inhibit cell proliferation and block the cell cycle at the G0/G1 phase and induce apoptosis, which can likely be ascribed to inhibition of NF-κB and STAT3 transcriptional activation [15]. PHB1 also has a strong anti-inflammatory effect, participating in the development and differentiation of immune cells [20, 21]. Our previous study showed that PHB1 was significantly downregulated in NPC tissues, and the decreased PHB1 expression correlated significantly with advanced clinical stage, metastasis and poor prognosis in NPC lesions, suggesting the expression level of PHB1 could be used as a potential prognostic bio-marker for NPC [22]. Considering the function of PHB1 in anti-tumour and anti-inflammation, PHB1 might play an important role in the initiation and progression of NPC. However, the effect and molecular mechanism of PHB1 and the relationship between PHB1 and LPLUNC1 requires further investigation in NPC.

In this study, we aimed to investigate the role of PHB1 in NPC and whether the anti-tumour role of LPLUNC1 is dependent on PHB1. Moreover, we found a possible mechanism of upregulation of PHB1 by LPLUNC1. This process may subsequently suppress the transcriptional activity of NF-κB, thus modulating cell proliferation and apoptosis in the development and progression of NPC. Our findings provide promising targets that could contribute to the diagnosis and treatment of NPC.

## Results

### LPLUNC1 upregulates PHB1 and is associated with PHB1 expression in NPC

**Fig. 1:**
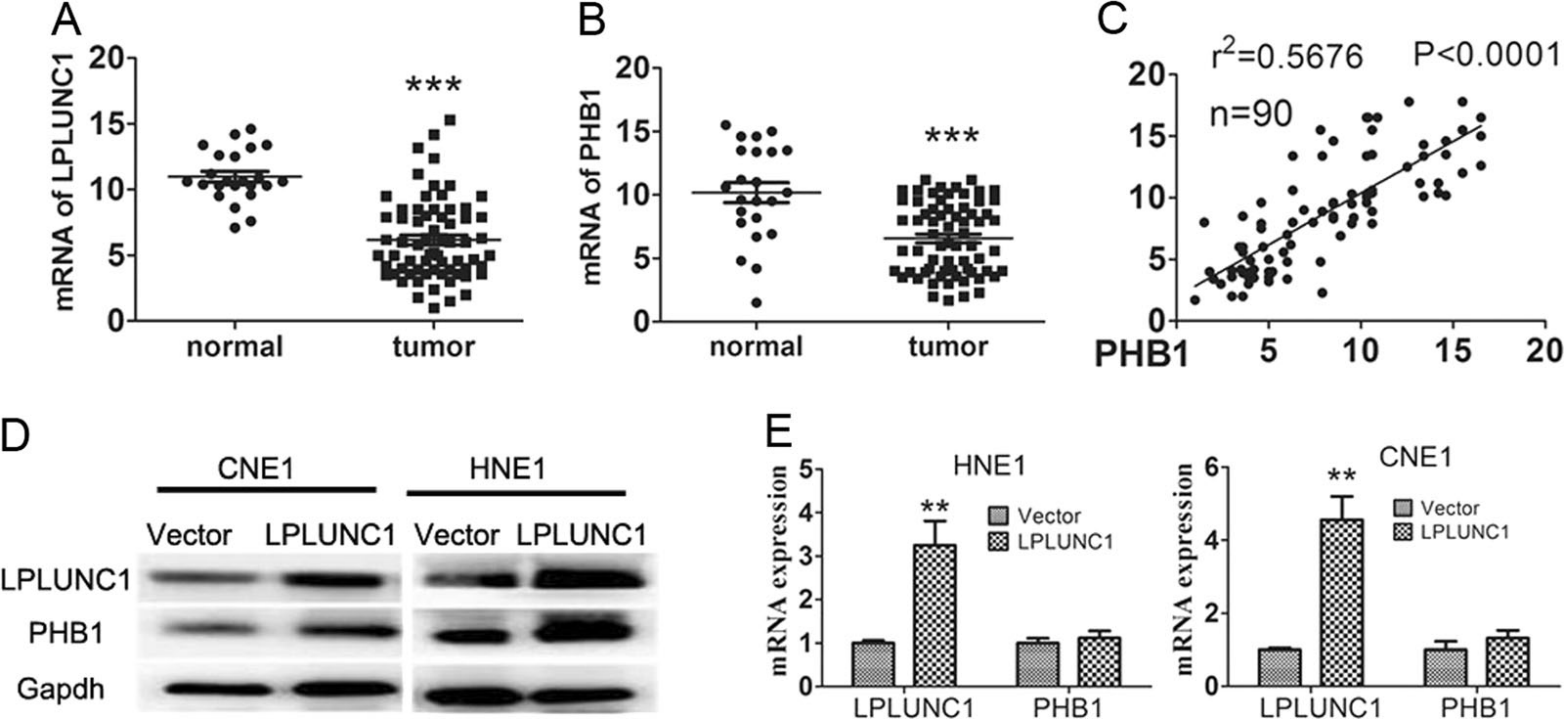
LPLUNC1 expression was positively correlated with PHB1 expression in NPC. **a** LPLUNC1 mRNA expression was analysed by qRT-PCR in normal and tumour tissues. **b** PHB1 mRNA expression was analysed by qRT-PCR in nasopharyngeal carcinoma (NPC, *N* = 67) specimens and normal nasopharyngeal epithelium (NPE, *N* = 23) specimens. **c** Linear Regression analysis of PHB1 and LPLUNC1 at mRNA level. *R* indicates correlation coefficient, analysed by Pearson Linear Regression, *p* < 0.00001. The protein (**d**) and mRNA (**e**) level of LPLUNC1 and PHB1 were detected by western blotting and qRT-PCR in the indicated cells. Data shown are representative images or expressed as the mean ± s.d. of each group from three separate experiments. (**P < 0.01. ***P < 0.001 vs. vector, Student’s *t*-test)

Our previous study found that LPLUNC1 upregulated PHB1 expression in NPC cells [13]. LPLUNC1 and PHB1 were significantly downregulated in NPC tissues, and the decreased LPLUNC1 and PHB1 expression significantly correlated with a poor prognosis of NPC [10, 22], which suggests a candidate tumour suppressor role in NPC. We re-analysed our previous immunhistochemistry results in which the same NPC samples were used to detect the LPLUNC1 and PHB1 expression [10, 22], and we found that the levels of LPLUNC1 protein expression were correlated positively with the levels of PHB1 protein expression in NPC specimens (Table S1 and Figure S1). To further confirm the relationship of LPLUNC1 and PHB1 in NPC tissues, we analysed the mRNA levels of LPLUNC1 and PHB1 in nasopharyngeal carcinoma (NPC) and normal nasopharyngeal epithelium (NPE) by qRT-PCR. As a result, most patients exhibited a significantly lower level of LPLUNC1 and PHB1 compared with the normal control tissues (Fig. 1a, b), as well a positive correlation between LPLUNC1 and PHB1 expression in NPC and normal samples analysed by Pearson Linear Regression (Fig. 1c, *P* < 0.0001). In addition, the high protein level of PHB1 was detected by Western blot in CNE1 and HNE1 cells with LPLUNC1 over-expression (Fig. 1d), but the PHB1 mRNA level was not affected (Fig. 1e). These results suggest that PHB1 may be regulated post-transcriptionally by LPLUNC1.

### LPLUNC1 upregulates PHB1 by blocking TRIM21-mediated ubiquitination

**Fig. 2:**
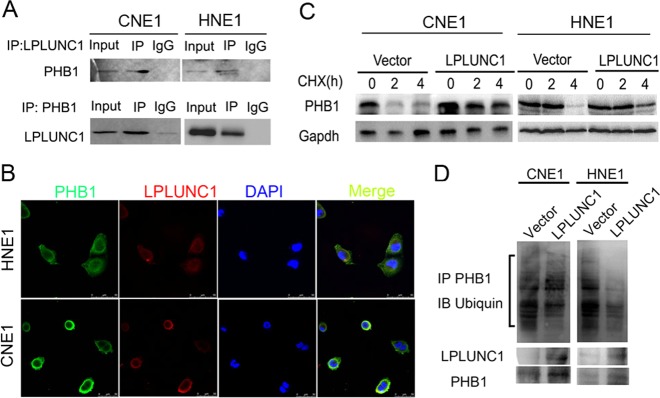
LPLUNC1 promoted PHB1 ubiquitination. **a** Co-IP analysis of interaction of LPLUNC1 and PHB1 in CNE1 and HNE1 cells. Normal mouse IgG was used as negative control. **b** PHB1 and LPLUNC1 expression were detected by immunofluorescence staining (Scale Bar, 10 μm). **c** Levels of PHB1 proteins in CNE1 and HNE1 cells with or without LPLUNC1 expression following 10 μg/ml CHX treatment for indicated times. **d** CNE1 and HNE1 cells were co-transfected with Flag-tagged PHB1, myc-tagged LPLUNC1 and HA-tagged ubiqutins. Cells treated with MG132 for 24 h, lysates of CNE1 and HNE1 cells were immunoprecipitated with Flag antibody and analysed by immunoblotting with anti-His antibody to examine ubiquitination of PHB1. Cell lysates were subjected to western blotting with an anti-LPLUNC1 or anti-PHB1 antibody (bottom) in whole-cell lysate. Data shown are representative images each group from three separate experiments

As LPLUNC1 is upregulated PHB1 at the protein level only, we hypothesised that there may be an interaction between LPLUNC1 and PHB1, and thus LPLUNC1 might affect the protein stability of PHB1. To validate the interaction of PHB1 with LPLUNC1, HNE1 and CNE1 NPC cells were co-transfected with Flag-PHB1 and Myc-LPLUNC1 and were subjected to Co-IP analysis with an anti-Flag antibody or anti-myc antibody. The results showed that exogenously expressed Flag-PHB1 specifically interacted with exogenously expressed myc-LPLUNC1 when co-expressed (Fig. 2a). The interaction of PHB1 and LPLUNC1 was confirmed by immunofluorescent co-localisation analysis (Fig. 2b). Next, we examined the protein stability of PHB1 in LPLUNC1 over-expressing cells and found that the degradation of PHB1 protein was significantly delayed by LPLUNC1 in the presence of protein synthesis inhibitor CHX (Fig. 2c), suggesting that LPLUNC1 could prevent the protein degradation of PHB1. It is known that ubiquitinated modification of proteins is usually involved in proteasome degradation [23, 24], so we examined whether LPLUNC1 affects the ubiquitination of PHB1. As shown in Fig. 2d, over-expression of LPLUNC1 significantly reduced the ubiquitination of PHB1 in NPC cells, suggesting that LPLUNC1 may block the ubiquitinated degradation of PHB1.

**Fig. 3:**
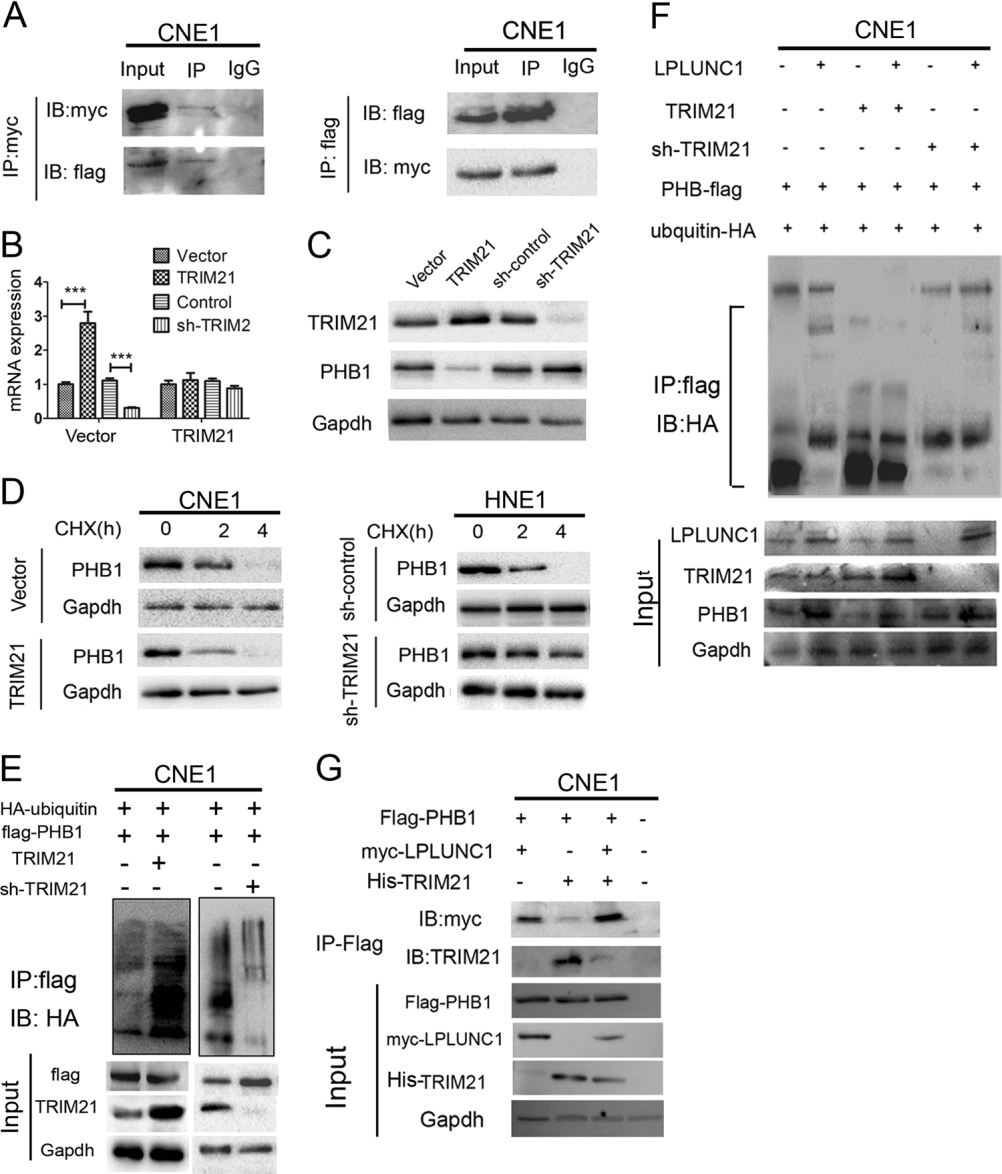
LPLUNC1 effects on TRIM21-meidated PHB1 ubiquitinating **a** exogenous PHB1 and TRIM21 proteins form stable complexes. Myc-tagged TRIM21 and Flag-tagged PHB1 were transiently over-expressed in CNE1 cells. At 48 h post-transfection, cells were harvested, and cell lysates were subjected to IP with antibody to Flag or Myc-tag, using normal mouse IgG (IgG) as a control. **b**, **c** The PHB1 expression in TRIM21 expression or silencing CNE1 cells was examined by qRT-PCR and western blotting. **d** Half-life of PHB1 protein is delayed by TRIM21. CNE1 and HNE1 cells were transfected with PCMV-TRIM21 or vector control. At 24 h post transfection, cells were treated with 100 μg/ml cycloheximide (CHX) for indicated time intervals, followed by IB analysis. **e** TRIM21 mediates poly-ubiquitination of PHB1 ubiquitination. CNE1 cells were transfected with Flag-tagged PHB1 and HA-ubiquitin, plus TRIM21, sh-TRIM21 or their vector controls. At 48 h post transfection, cells were treated with MG132 for 4 h, and then lysates of these cells were subjected to IP with anti-Flag-conjugated beads. Immunoprecipitates were subjected to IB analysis for ubiquitinated PHB1 with antiubiquitin or for indicated proteins. **f** CNE1 cells were transfected with Flag-tagged PHB1 and HA-ubiquitin, plus TRIM21, sh-TRIM21, LPLUNC1 or their vector controls. At 48 h post transfection, cells were lysed and subjected to IP with anti-Flag-conjugated beads. The immunoprecipitates were subjected to IB analysis for ubiquitinated PHB1 with antiubiquitin or for indicated proteins. **g** IP analysis using anti Flag antibody followed by IB analysis to detect LPLUNC1 and TRIM21 expression in CNE1 cells. Data shown are representative images or expressed as the mean ± s.d. of each group from three separate experiments. (***P < 0.001 vs. controls)

Since LPLUNC1 is not an E3 ligase, we wondered how LPLUNC1 affects the ubiquitination of PHB. Interestingly, when we identified the proteins that interacted with LPLUNC1 by performing Co-IP and mass spectrometry assays, we found that TRIM21 was an interaction protein of LPLUNC1 (data not shown). As TRIM21 is a RING-finger domain-containing ubiquitin E3 ligase that plays an important role in cancer progression [25–27], we hypothesised that TRIM21 might be involved in the blockage of the ubiquitinated degradation of PHB1 by LPLUNC1. We further validated the interaction of PHB1 and TRIM21 in NPC cells and found that TRIM21 can interact with PHB1 (Fig. 3a), suggesting TRIM21 may be the E3 ligase responsible for polyubiquitination of PHB1. To determine whether TRIM21 is the E3 ligase responsible for PHB1 ubiquitination, we investigated the effects of TRIM21 on PHB1 mRNA and protein levels, half-life and ubiquitination. We found that knockdown of TRIM21 increased PHB1 protein levels, but not PHB1 mRNA levels, while ectopically expressing TRIM21 decreased PHB1 protein levels, but not PHB1 mRNA levels (Fig. 3b, c). Moreover, TRIM21 over-expression resulted in a shorter PHB1 protein half-life, while knockdown of TRIM21 prolonged PHB1 protein half-life (Fig. 3d). Ubiquitination assays showed that TRIM21 expression increased PHB1 polyubiquitination levels in the presence of the specific proteasome inhibitor, MG132, while down-regulation of TRIM21 decreased PHB1 ubiquitination levels in CNE1 cells (Fig. 3e). These data indicate that TRIM21 is the E3 ligase responsible for ubiquitination of PHB1.

As TRIM21 is an E3 ubiquitin ligase that acts on PHB1, and LPLUNC1 inhibits PHB1 ubiquitination and degradation, we further surmised whether LPLUNC1 regulates the TRIM21-mediated ubiquitination of PHB1. We first evaluated the effects of LPLUNC1 on TRIM21 expression. The results showed that LPLUNC1 overexpression had no significant effects on TRIM21 both at the mRNA and protein levels. TRIM21 also had no effects on LPLUNC1 at either the mRNA and protein levels (Fig. S2). However, we observed that LPLUNC1 significantly inhibited TRIM21-mediated PHB1 ubiquitination (Fig. 3f), suggesting that LPLUNC1 could affect TRIM21-mediated PHB1 ubiquitination. As LPLUNC1 and TRIM21 can both interact with PHB1 and affect the ubiquitination of PHB1, we wondered what the mechanisms in the process? Thus, flag-tagged PHB1, myc-tagged LPLUNC1 and his-tagged TRIM21 were co-expressed in CNE1 cells. An immunoprecipitation assay showed that PHB1 had a strong interaction with LPLUNC1 but a weak interaction with TRIM21 (Fig. 3g), suggesting that LPLUNC1 competitively interacted with PHB1, disrupted the binding of TRIM21 to PHB1, and thereby inhibited PHB1 ubiquitination, upregulating PHB1 expression.

### PHB1 acts as a tumour suppressor by inhibition of NF-κB activity

**Fig. 4:**
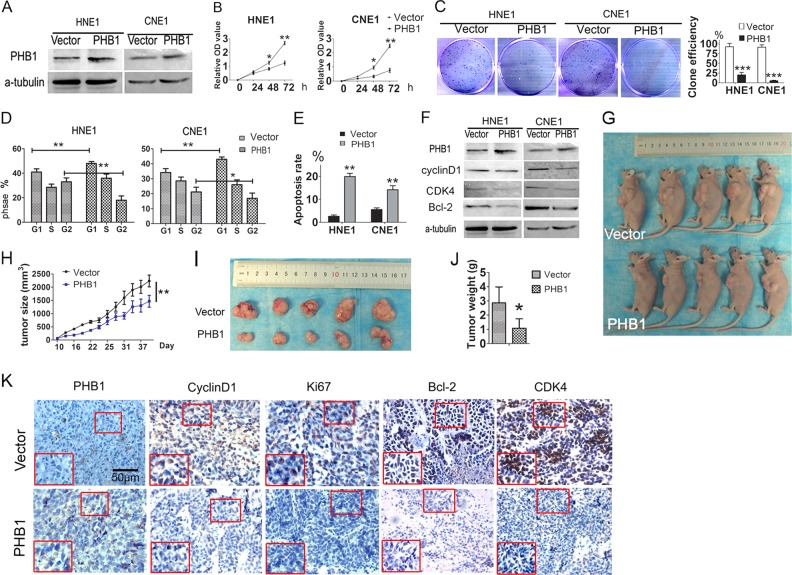
PHB1 inhibited cell proliferation and promoted apoptosis of CNE1 and HNE1 cell lines. **a** Western blotting analysis of PHB1 expression in PHB1 stably over-expressing CNE1 and HNE1 cells. **b** Cck-8 assays of CNE1 and HNE1 cells transfected with PHB1 over-expressing vector (PHB1) or negative control vector (Vector). Error Bar = s.d. *p < 0.05, **p < 0.01 compared to vector, independent Student’s *t-*test). **c** Colony-forming assay images (left panel) and quantification of colony number/well (right panel). Error bar = s.d. **p < 0.001 compared to vector, independent Student’s *t-*test). **d** Quantification of cell-cycle analysis by flow cytometry. Error Bar = s.d. *p < 0.05, **p < 0.01 compared to vector, independent Student’s *t-*test). **e** Quantification of Annexin V-FITC and PI double staining analysis of cell apoptosis by flow cytometry. **f** Expression of apoptosis marker Bcl2, cell cycle markers cyclin D1, and CDK4 were determined by western blotting. α-Tubulin served as an internal control. **g** Images of CNE1 xenograft model in nude mice (*n* = 5 per group). **h** Tumour growth curve in xenograft model at indicated time. Error Bar = s.d. **p < 0.01 compared to vector, independent Student’s *t-*test. **i**, **j** Tumour images and weight quantification of tumours. Error Bar = s.d. *p < 0.05 compared to vector, independent Student’s *t-*test. **k** IHC for quantification of proliferation marker Ki-67 and cell cycle marker CyclinD1 and CDK4, apoptosis marker Bcl-2. Scale bar is 50 μm. Data shown are representative images or expressed as the mean ± s.d. of each group from three separate experiments (in vitro) or one separate experiment (in vivo). (*P < 0.05, **P < 0.01, ***P < 0.001 vs. vector, Student’s *t* test)

Our previous study showed that LPLUNC1 is a potential tumour suppressor in NPC [9, 10]. As a downstream target of LPLUNC1, we wondered whether PHB1 functions as a tumour suppressor. We found that PHB1 expression levels were lower in NPC cells CNE1, CNE2, HNE1, HNE2, 5–8F than in normal nasopharyngeal epithelial cells NP69 (Figure S3A and 3B). To elucidate the function of PHB1 in NPC, we established PHB1 stable expression in CNE1 and HNE1 cell lines, as confirmed by Western blotting (Fig. 4a). As shown in Fig. 4b, c, we found that PHB1 overexpression significantly suppressed cell viability and colony formation, and obviously induced G1/S arrest and apoptosis in these cells (*P* < 0.001, Fig. 4d, e, Figure S2C and S2D). PHB1 overexpression significantly decreased the levels of cyclin D1, CDK4, and Bcl-2 (Fig. 4f). We further examined the role of PHB1 in xenografts of nude mice, and the results showed that PHB1 over-expression obviously suppressed the growth of xenografts in nude mice compared to the control group (*P* < 0.001, Fig. 4g, h). The average weight of PHB1 over-expression tumours was significantly lower than that of the control group (*P* < 0.001, Fig. 4i, j). IHC analyses revealed that high levels of PHB1 were expressed in the PHB1 over-expression group, accompanied by lower-intensity anti-Ki67, anti-cyclin D1, anti-CDK4, and anti-Bcl-2 staining, indicating low levels of NPC cell proliferation (Fig. 4k).

**Fig. 5:**
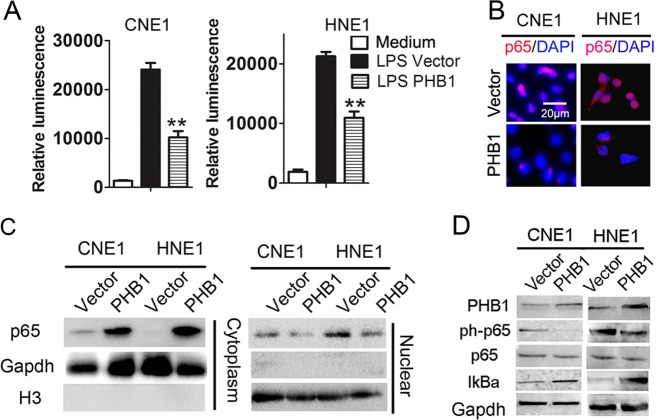
PHB1 inhibited LPS-induced NF-κB activation in NPC cells. **a** After LPS (1 µg/ml) treatment for 1 h, NF-κB reporter activity following PHB1 overexpression in CNE1 and HNE1 cells. **b** Immunofluorescence microscopy detection of p65 (red) and nuclei (blue) of CNE1 and HNE1 cells. **c** Western blotting analysis of p65 in nuclear and cytoplasmic fractions following PHB1 over-expression in CNE1 and HNE1 cells. **d** Western blot analysis of NF-κB signal pathway following PHB1 over-expression in CNE1 and HNE1 cells. **e** Tumours in nude mice were analysed per group using immunohistochemistry for molecules mentioned above. Scale bar is 20 μm. Data shown are representative images or expressed as the mean ± s.d. of each group from three separate experiments. (**P < 0.01 vs. vector, Student’s *t-*test)

Deregulated activation of NF-κB is widespread in human cancers, promoting the survival of tumour cells [28–30]. We examined whether PHB1 could inhibit NF-κB activation in NPC cells, even after LPS stimulation. Dual-luciferase reporter assays revealed that significantly lower levels of NF-κB transcription activity were detected in the PHB1 over-expression of NPC cells, compared to control cells, even after LPS stimulation (Fig. 5a). Similarly, there was lower-intensity anti-phospho-NF-κB p65 staining in the nuclei of PHB1 over-expression NPC cells, even after LPS stimulation (Fig. 5b). Western blot analysis also showed that the level of phosphorylated-NF-κB p65 in PHB1 over-expression NPC cells were obviously lower than that in the control cells in both the absence and presence of LPS (Fig. 5c), accompanied by upregulation of IκBα expression (Fig. 5d). These results suggest that PHB1 can act as a tumour suppressor via inhibition of the NF-κB signalling pathway.

### LPLUNC1 suppresses NPC cell proliferation partly through a PHB1-mediated mechanism

**Fig. 6:**
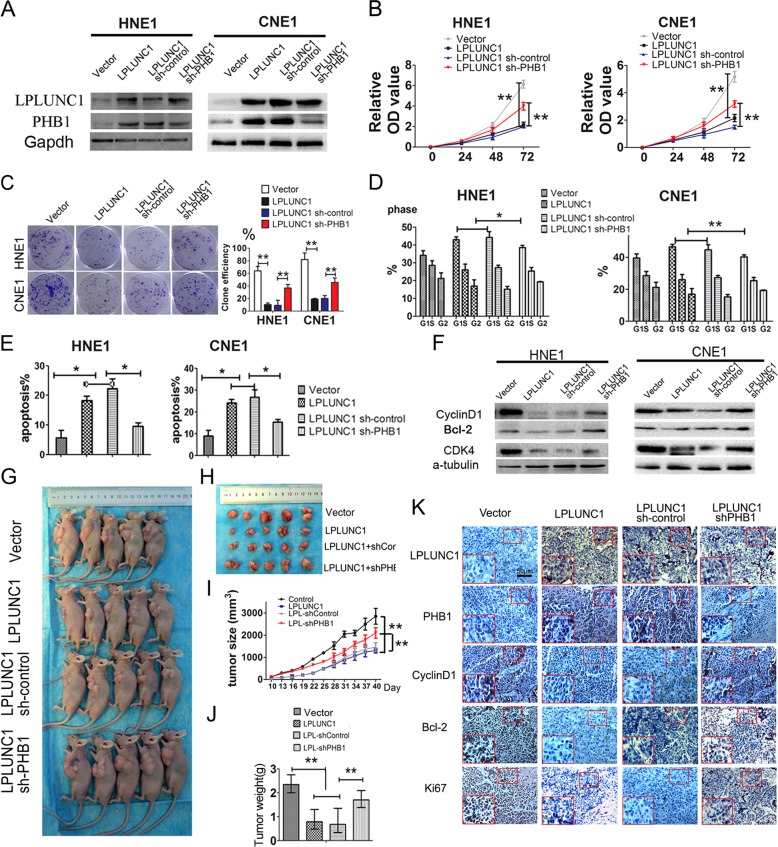
**a** LPLUNC1 inhibited cell proliferation and promoted apoptosis by upregulating PHB1 expression in CNE1 and HNE1 cells. **b** CCk-8 assays of cells transfected with LPLUNC1 and control vector, LPLUNC1 and shPHB1 in combination. **c** Colony-forming assay images (left panel) and quantification of colony number/inoculated number (right panel). **d** Cell-cycle analysis by flow cytometry. **e** Annexin V-FITC and PI double staining analysis of cell apoptosis by flow cytometry. **f** Expression of apoptosis marker Bcl-2 and cell cycle markers cyclinD1 and CDK4 were determined by western blot. α-Tubulin served as an internal control. **g** Images of CNE1 xenograft model in nude mice. **h** Tumour growth curve at indicated time. **i**, **j** Tumour images and weight quantification of tumours (**k**) IHC for quantification of proliferation marker Ki-67 and image apoptosis marker Bcl-2 for quantification of apoptosis as well as cell cycle markers Cyclin D1 and CDK4. Scale bar is 50 μm. Data shown are representative images or expressed as the mean ± s.d. of each group from three separate experiments (in vitro) or one separate experiment (in vivo). (*P < 0.05, **P < 0.01 vs. controls, Student’s *t*-test or two-way ANOVA)

Based on the relationship of LPLUNC1 and PHB1, we further investigated the mechanisms underlying the action of LPLUNC1. We examined whether the tumour suppression of LPLUNC1 requires PHB1 expression. We used short-hairpin RNA to knock down the expression of PHB1 in HNE1 and CNE1 cells with LPLUNC1 over-expression and examined the tumour suppressive effects of LPLUNC1. The expression of LPLUNC1 and PHB1 was confirmed in cells transfected with LPLUNC1 and shPHB1 (Fig. 6a). Knockdown of PHB1 significantly promoted the cell proliferation and colony-formation of HNE1 and CNE1 cells compared to respective controls (Fig. 6b, c). Furthermore, knockdown of PHB1 could cause a significant increase in the G0/G1 population, with a decrease in the S-phase or G2/M in LPLUNC1-overexpressing CNE1 and HNE1 cells, respectively (Fig. 6d, Figure S4A). Knockdown of PHB1 also significantly attenuated the inhibition of LPLUNC1 on the apoptosis in CNE1 and HNE1 cells (Fig. 6e, Figure S4B). Compared to the LPLUNC1 control group, knockdown of PHB1 significantly increased the expression of cyclinD1, CDK4, and Bcl-2 as measured by western blot (Fig. 6f).

Next, we detected the effects of PHB1 silencing on the tumour suppression of LPLUNC1 in vivo. The results showed that PHB1 silencing significantly promoted the subcutaneous tumour growth of LPLUNC1 over-expression NPC cells (Fig. 6g, h). The weights of shPHB1/LPLUNC1 group tumours were significantly larger than those of the sh-RNA control or LPLUNC1 group (Fig. 6i, j). IHC analyses revealed the levels of LPLUNC1 and PHB1 in the tumour xenografts (Fig. 6k). In concordance with the results in vitro, PHB1 silencing significantly increased the Ki67, cyclin D1 and Bcl-2 expression compared to sh-RNA control or LPLUNC1 group (Fig. 6k). Collectively, our findings in vitro and in vivo suggest that PHB1 silencing can attenuate the tumour suppressor function of LPLUNC1 in NPC, indicating that the tumour suppressive role of LPLUNC1 partly requires PHB1 expression in NPC cells.

**Fig. 7:**
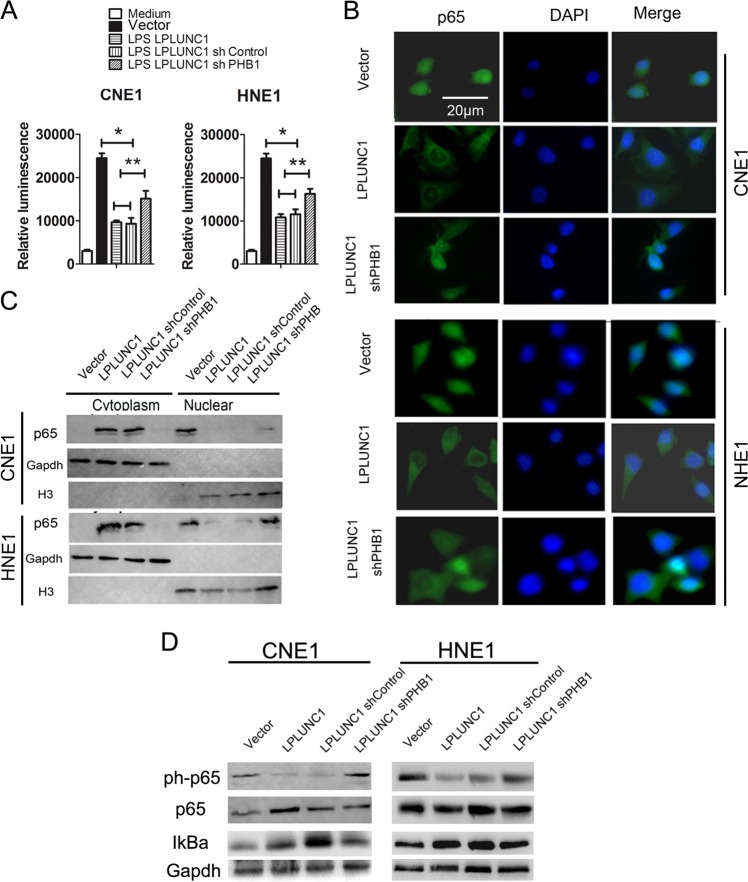
LPLUNC1 inhibited LPS-induced NF-κB activation through upregulation of PHB1 in NPC cells. **a** After LPS (1 µg/ml) treatment for 1 h, NF-κB reporter activity following PHB1 over-expression in CNE1 and HNE1 cells. **b** immunofluorescence microscopy detection of p65 (red) and nuclei (blue) of CNE1 and HNE1 cells. **c** Western blotting analysis of p65 in nuclear and cytoplasmic fractions following PHB1 over-expression in CNE1 and HNE1 cells. **d** Western blotting analysis of NF-κB signal pathway following PHB1 over-expression in CNE1 and HNE1 cells. **e** Tumours in nude mice were analysed per group using immunohistochemistry for molecules mentioned above. Scale bar is 20 μm. Data shown are representative images or expressed as the mean±s.d. of each group from three separate experiments. (*P < 0.05, **P < 0.01 vs. controls, two-way ANOVA)

Furthermore, we analysed whether LPLUNC1 could inhibit NF-κB activation, which is mediated by PHB1 in NPC cells. Our results showed that NF-κB activity was significantly reduced in the LPLUNC1 group, even after LPS stimulation, while the inhibition of LPLUNC1 on NF-κB activity was obviously reversed by knockdown of PHB1 (Fig. 7a). Similarly, there was less intensity of anti-phospho- NF-κB p65 staining in the nuclei of LPLUNC1 over-expression NPC cells, even after LPS stimulation, but the inhibition of LPLUNC1 was attenuated by knockdown of PHB1 (Fig. 7b, c). Western blot analysis also showed that the level of phosphorylated-NF-κB p65 in LPLUNC1 overexpression NPC cells was obviously lower than in control cells in both the absence and presence of LPS, accompanied by upregulation of IκBα expression, while the effect of LPLUNC1 on NF-κB p65 and IκBα was obviously reversed by knockdown of PHB1 (Fig. 7d). These results suggest that LPLUNC1 can inhibit the activation of NF-κB signalling pathway by inducing PHB1 expression in NPC.

## Discussion

Three PLUNC proteins (SPLUNC1, SPLUNC2 and LPLUNC1) were found in mucoepidermoid carcinomas and papillary cystadenocarcinoma but were not detected in other tumours [31]. Long-palate, lung and nasal epithelium clone 1 (LPLUNC1) is specifically expressed in nasopharyngeal epithelial tissue and the trachea [32] but is significantly down-regulated in nasopharyngeal carcinoma [9]. Our previous studies showed that LPLUNC1 inhibited NPC cell proliferation in vitro and in vivo. LPLUNC1 also delayed cell cycle progression from G1 to S phase and inhibited the expression of cyclin D1, cyclin-dependent kinase 4 (CDK4) and phosphorylated Rb [9, 33]. LPLUNC1 also markedly inhibited NPC cell migration, invasion, and lung-metastasis [11, 12]. Despite its basic biological and clinical importance, the underlying mechanisms of LPLUNC1 remain poorly understood. In our previous study, we established a differential proteins expression profile in LPLUNC1 over-expression NPC cells using 2-D DIGE with MALDI-TOF-MS/MS, and among these differentially expressed proteins, PHB1 was upregulated by LPLUNC1 [13]. Moreover, LPLUNC1 and PHB1 were significantly downregulated in NPC tissues, and the decreased LPLUNC1 and PHB1 expression significantly correlated with a poor prognosis in NPC. In this study, we further confirmed that LPLUNC1 and PHB1 were significantly downregulated at the mRNA level in NPC tissues, and there was a significantly positive correlation between LPLUNC1 and PHB1 expression in NPC, suggesting that PHB1 was involved in the role of LPLUNC1 in suppression of NPC.

PHB1 was originally cloned from regenerating livers and was thought to be a tumour suppressor (hence its name). Numerous studies showed that PHB1 is a potential target for cancer therapy [34, 35]. A clear tumour suppression role of PHB1 has been demonstrated in breast, gastric and prostate cancers [36–39]. In human hepatocellular carcinoma (HCC) and cholangiocarcinoma (CCA) cells, PHB1 inhibits cell growth and negatively regulates the expression of oncogenes c-MYC, MAFG, and c-MAF. Liver-specific Phb1 knockout mice showed a high ability to form spontaneous hepatocellular carcinoma [40]. In the current study, we found that PHB1 over-expression significantly inhibited the proliferation of NPC cells in vitro and in vivo, suggesting that PHB1 is a tumour suppressor gene in NPC. Interestingly, we also found that deficiency in PHB1 could reverse the inhibition of LPLUNC1 on cell proliferation and apoptosis in vitro and in vivo. These results suggest a mechanism in which LPLUNC1 functioned as a tumour suppressor through upregulation of PHB1 in NPC.

As LPLUNC1 did not significantly upregulate PHB1 on mRNA level, LPLUNC1 might regulate PHB1 via post-translational modifications. Interestingly, we treated LPLUNC1 over-expressing cells with CHX (a protein synthesis inhibitor), and found that the degradation of PHB1 protein was significantly delayed by LPLUNC1, suggesting that PHB1 stability could be enhanced by LPLUNC1. The ubiquitination system functions in a wide variety of cellular processes, which include different types of substrate ubiquitination, such as the addition of a single ubiquitin molecule (monoubiquitination) or various ubiquitin chains (polyubiquitination) [41]. Whether LPLUNC1 participates in monoubiquitination or polyubiquitination is still to be investigated. In this study, we found that there was an interaction of PHB1 and LPLUNC1, and over-expression of LPLUNC1 significantly reduced the ubiquitination of PHB1 in NPC cells, suggesting that LPLUNC1 could block the ubiquitinated degradation of PHB1. However, the domains of binding for each must be further confirmed. Therefore, our results showed that LPLUNC1 stabilised PHB1 protein though inhibition of PHB1 ubiquitination, which delays the degradation of PHB1 in NPC cells. These data suggest that LPLUNC1 functions as a tumour suppressor by stabilising PHB1 protein and inhibition of tumour growth.

PHB1 ubiquitination has been identified in spermatogenesis [42]. However, the mechanism of PHB1 ubiquitination has not been clarified. TRIM21 is a RING-finger domain-containing ubiquitin E3 ligase that plays an important role in cancer progress [25, 43]. In our study, we found that TRIM21 (one interaction protein of LPLUNC1) could interact with PHB1 and promoted PHB1 ubiquitinated degradation, indicating that TRIM21 is the E3 ligase responsible for ubiquitination of PHB1. Furthermore, we found that LPLUNC1 significantly inhibited the TRIM21-mediated PHB1 ubiquitination, and further found that PHB1 had a strong interaction with LPLUNC1 but a weak interaction with TRIM21. Collectively, our results suggest LPLUNC1 competitively interacts with PHB1 and disrupts the binding of TRIM21 to PHB1, thus inhibiting PHB1 ubiquitination and upregulating PHB1 expression. However, the detailed mechanism of LPLUNC1 inhibition of PHB1 ubiquitination must be further investigated. This is the first time that it has been demonstrated that TRIM21 mediates the ubiquitination of PHB1. TRIM21 may be the E3 ligase of PHB1 ubiquitination degradation, and LPLUNC1 upregulates PHB1 by blocking TRIM21-meidated PHB1 ubiquitination.

Our previous studies have demonstrated that LPLUNC1 might suppress NPC cell proliferation via inhibiting the Stat3 activation [10] or the mitogen-activated protein (MAP) kinase and cyclin D1/E2F pathways [9], and reducing NPC cell migration and invasion by interacting with VTN or VIM through suppressing the FAK-signalling pathway by disassociating the VTN/ITGAV complex [11]. LPLUNC1 also regulates the NPC cell radioresponse by inhibiting VTN-induced activation of the ataxia telangiectasia mutated kinase-Chk2 (ATM-Chk2) and ataxia telangiectasia and Rad3-related kinase-Chk1 (ATR-Chk1) pathways after ionising radiation (IR) [12]. However, the molecular mechanism of action of LPLUNC1 is still unclear in NPC.

Given to NPC is an inflammation-related cancer [3], LPLUNC1 and PHB1 function in anti-tumour and anti-inflammation [33, 44], and we demonstrated that LPLUNC1 inhibited NPC growth by suppressing the Stat3 pathway [10]. In addition, PHB1 has a strong anti-tumour and anti-inflammatory effect via blocking NF-κB and STAT3 signalling pathway [45–47]. NF-κB and STAT3 signalling pathways collaboratively link inflammation to cancer [48]. In this study, we examined whether LPLUNC1 could inhibit NF-κB activation mediated by PHB1 in NPC cells, even in LPS stimulation. We found that respective upregulation of LPLUNC1 and PHB1 obviously inhibited activation of the NF-κB pathway, even by LPS stimulation, but inhibition of LPLUNC1 on blockage of the NF-κB pathway was significantly reversed by knockdown of PHB1, and thus LPLUNC1 can inhibit activation of the NF-κB signalling pathway mediated by PHB1, consequently contributing to inhibition of the growth of NPC.

In summary, our current work shows an important role for LPLUNC1 and PHB1 in NPC. LPLUNC1 induced PHB1 expression via inhibition of PHB1 ubiquitination as mediated by TRIM21. PHB1 acted as a tumour suppressor gene by inhibition of NF-κB activity. Depletion of PHB1 rescued the anti-tumour effects of LPLUNC1, which suggested that LPLUNC1 inhibited NPC cell proliferation partly through a PHB1-mediated mechanism. Furthermore, LPLUNC1 inhibited activation of the NF-κB signalling pathway via induction of PHB1 expression in NPC, even after LPS stimulation. Together our data suggest that LPLUNC1 exerts a tumour-suppressive role by downregulating the NF-κB pathway via inhibition of PHB1 ubiquitination and degradation mediated by TRIM21. PHB1 may also represent a promising candidate tumour suppressor gene associated with NPC. Our findings further revealed a novel mechanism underlying the anticancer effect of LPLUNC1 and clarified that PHB1 was a tumour suppressor in NPC and provide potential targets for NPC diagnosis and treatment.

## Material and method

### Patients and tissue samples

A total of 67 NPC specimens were collected from patients with NPC who underwent curative tumour resection at Hunan cancer hospital (Changsha, China) between January 2013 and 2015. During surgery, a total of 23 adjacent non-tumourous nasopharyngeal tissues were collected as controls. No patients underwent chemotherapy or radiotherapy prior to surgery. All experimental procedures were approved by the ethics committee of the Affiliated Cancer Hospital of Xiangya School of Medicine, Central South University and Hunan Cancer Hospital (Changsha, China), and an informed consent was obtained from all patients. The clinical stage of the patients was classified according to the Pathology of nasopharyngeal carcinoma by WHO [49]. The clinicopathological information for all patients is presented in Table 1.

**Table 1:** Clinicopathologic characteristics

Characteristics	Cases
*Normal*	23
Age	
≤48	10
>48	13
Gender:	
male	17
female	6
*Tumour*	67
Age	
≤48	29
>48	38
Gender:	
male	49
female	18
Metastasis	45
No metastasis	21
Stages I + II	27
Stages III + IV	40

### Cell lines

The human NPC cell lines CNE1, HNE1, CNE2, HNE2, 5-8 F, NP69 were maintained in our lab, which used in our previous study [10, 13]. The cells were cultured in RPMI-1640 (Gibco, Logan, UT, USA) supplemented with 10% foetal bovine serum (FBS; Zeta, South Africa) in a humidified incubator with 5% CO2 at 37 °C. All cells were routinely tested for negative mycoplasma contamination using Mycoplasma Detection Kit (Sigma). The expression vector of PHB1, TRIM21 and LPLUNC1 and short hairpin RNA for PHB1 and TRIM21 were purchased from Genechem Co., LTD. For stable gene transfection, HNE1 and CNE1 cells were seeded overnight and then transfected with a PHB1-GV143-neo expression vector, a LPLUNC1-PIRES-neo expression vector and a sh-PHB1-GV102-neo expression vector, as well as a control vector for selection of neomycin-resistant cells. After a month, a pool of neomycin-resistant tumour cells (>8 clones) was selected and confirmed by western blot analysis for PHB1 or LPLUNC1 expression.

### Western blotting and co-immunoprecipitation

Cells and tissue samples were lysed in RIPA buffer in the presence of Protease Inhibitor Cocktail and PhoSTOP (Roche, Basel, Switzerland). Protein was quantified using a BCA Protein Assay Kit (Pierce Biotechnology, Rockford, IL, USA). Protein (30 μg) was separated using 10% sodium dodecyl sulphate polyacrylamide gel electrophoresis and transferred onto polyvinylidene fluoride membranes (PVDF) (Millipore, Billerica, MA, USA). The membranes were blocked with 5% non-fat milk in Tris-buffered saline and then incubated with primary antibodies at 4 °C overnight. The primary antibodies used were available in supplementary Table S2. Membranes were washed three times in TBST solution for 10 min each and then incubated with secondary antibodies. Signals were detected by an enhanced chemiluminescence detection system as the manufacturer’s protocol (Bio-Rad, Hercules, CA, USA). For Co-IP, proteins were immunoprecipitated by anti-PHB1 or LPLUNC1 antibody and then subjected to western blotting with antibody to LPLUNC1 and PHB1.

### RNA isolation and real-time PCR

Total RNA was isolated using Trizol (Invitrogen, CA) from NPC cell lines. Gene expression was assessed using real-time PCR. Total RNA was subjected to reverse transcription (RT) using Start Fast Reverse transcriptase (themo, the Universal PCR Master Mix were purchased from Bio-Rad (Hercules, CA). Hypoxanthine phosphoribosyltransferase 1 or glyceraldehyde 3-phosphate dehydrogenase (GAPDH, for human CCA) was used as a housekeeping gene. The thermal profile consisted of an initial denaturation at 95 °C for 3 min followed by 40 cycles at 95 °C for 3 sec and at 60 °C for 30 sec. The cycle threshold (Ct value) of the target genes was normalised to that of the housekeeping gene to obtain the delta Ct (Ct). The Ct was used to find the relative expression of target genes according to the formula: relative expression = 2^−^^Ct^, where Ct = Ct of target genes experimental condition Ct of target gene under control condition. The average of three independent analyses for each gene was calculated. Quantitative real-time PCR (qRT-PCR) was conducted using custom-made primers as follows: Gapdh, forward 5′-GAAGGTGAAGGTCGGAGTC-3′ and reverse 5′-GAAGATGGTGATGGGATTTC-3′; and PHB, forward 5′-CGGAGAGGACTATGATGAG-3′ and reverse 5′-GGTCAGATGTGTCAAGGA-3′ and LPLUNC1, forward 5′-CTTCCTGGTGAACGCCTTAG-3′ and reverse 5′-AAACTCCAGACGGTCAATGC-3′ and TRIM21, forward 5′-AGAATCCTGGGGGAGAAAGA-3′ and reverse 5′-TGGCACACACTCCTGAGTTC-3′

### Cck-8 assay and colony formation assay

The Cck-8 assay was used to detect the proliferation rate of tumour cells. Cells of CNE1 and HNE1 were stably transfected with vector expressing the PHB1, LPLUNC1, LPLUNC1 and shRNA expressing shPHB1 in combination, and their control vectors, respectively. Cells were trypsinized and seeded in 96-well plates at a density of 5 × 10^3^ cells/well in 200 μL of complete medium. Then, 10 μL of 5 mg/mL CCK-8 (Sigma, USA) was placed in each well at 0, 24, 48, and 72 h and incubated at 37 °C for 4 h. The absorbance was measured at 460 nm. For the colony-forming assay, ~300 cells per well were added to a 6-well plate, with three wells per sample. After a 14-day incubation, the cells were washed twice with PBS and stained with Giemsa solution (Beyotime, Beijing, China). Colonies containing more than 50 cells were counted as one positive colony. The number of colonies was calculated by Image Pro Plus 6.0. The plate clone formation efficiency was calculated as (number of colonies/number of cells inoculated)×100%. All experiments were performed in triplicate.

### Cell cycle and cell apoptosis assays

The cells were trypsinized, washed with PBS (phosphate buffered saline), and fixed in 70% ethanol at 4 °C overnight. Cell cycle analysis was prepared using a cell cycle detection kit (BD Corp., USA). After that, the cells were incubated in propidium iodide (PI) at 37 °C for 30 min in the dark. The cell cycle was analysed by flow cytometer (BD FACSVerse, USA). For cell apoptosis analysis, cells were washed twice with cold BioLegend’s Cell Staining Buffer, and then were resuspended in Annexin V Binding Buffer at a concentration of 0.25–1.0×10^7^ cells/ml. Then, 100 µl of cell suspension was transferred to a 1.5-ml test tube with 5 µl of FITC Annexin V and 10 µl of Propidium Iodide Solution. The cells were gently vortexed and incubated for 15 min at room temperature (25 °C) in the dark. Then, 400 µl of Annexin V Binding Buffer was added to each tube, followed by analysis by flow cytometry with the proper machine settings.

### Analyses of protein degradation

Analyses of protein degradation at 24 h after transfection, CNE1 and HNE1 cells were treated with 100 μg/ml cycloheximide and harvested at various time points. The cell lysate was subjected to immunoblotting. For analysis of protein ubiquitination of PHB1, cells were co-transfected with flag-tagged PHB1 and myc-tagged LPLUNC1 and HA-tagged ubiquitins. At 24 h after transfection, the proteasome inhibitor MG132 (10 μM) was added and the cells were incubated for 6 h. An anti-flag antibody was added to the cell lysates before incubation overnight at 4 °C. Protein A/G Plus–agarose was added and the mixture was incubated for 4 h at 4 °C. The ubiquitins were detected using HA antibody.

### NF-κB activity assays

The cells were seeded in a 24-well plates. After 24 h, the cells were transfected with NF-κB activity reporter vector, together with the pRL-TK vector (Promega) containing Renilla luciferase. Transfection was performed using Lipofectamine 3000 (Invitrogen). Cells were harvested 36 h post transfection. Firefly and Renilla luciferase activities were measured using a Dual-luciferase reporter kit (Promega) according to the manufacturer’s protocol. Firefly luciferase activity was normalised to Renilla luciferase activity.

### Immunofluorescence and fluorescence microscopy

Cells were grown on glass coverslips as previously described and incubated with antibodies for PHB1, LPLUNC1 and p65, followed by incubation with secondary antibodies (Supplymentary Table S2). After incubation with DAPI for 10 min at room temperature to stain the nuclei, cells were imaged using a fluorescent microscope (AX10 VERT A1, CarlZeiss AG, Germany) or confocal laser scanning microscope (Ultra-View Vox; Perkin-Elmer, Waltham, MA, USA).

### In vivo tumorigenicity assay

Considering the effect size and standard deviation, the method for determining the sample size in animal study is recommended by animal research committeese. Female 7-week-old, nu/nu-BALB/c athymic nude mice were acclimated in the animal house 1 week before experimentation. Six female BALB/c nude mice (7-week-old) were randomly divided into 6 groups, with 10 mice in each group. Then, 5 × 10^6^ cells resuspended in the total volume of 100 μl transfected with PHB1, LPLUNC1, LPLUNC1 and shPHB1 in combination and their control groups were implanted subcutaneously into each mouse from each group. Tumour growth was analysed by calliper measurements every 3 days and calculated using the formula 1/2 (length (mm)) × (width (mm))^2^. After the mice were killed, primary tumours were excised and formalin-fixed. Samples were paraffin embedded, cut at 4 μm and immunohistochemistry-stained for histological evaluation of target protein expression. The primary antibody (Table S2) was subject to IHC analysis.

### Immunohistochemistry

Standard immune-staining was used for immunohistochemical staining and PHB1 and LPLUNC1 protein expression in NPC and NPE samples were evaluated. Samples were observed using an Olympus microscope IX51 (Olympus, Tokyo, Japan). Two pathologists evaluated the immunohistochemical staining according to the blind principle. Each section was semiquantitatively scored based on the extent and intensity of immunoreactivity: 10% immunoreactive cells scored 0, 25% immunoreactive cells scored 1, 25–75% immunoreactive cells scored 2 and >75% immunoreactive cells scored 3. In addition, staining intensity was graded on a 0–3 scale: negative graded 0, weak graded 1, intermediate graded 2 and strong graded 3. The final score was designated as the sum of extent and intensity and samples categorised as low-expression (0–2) and high expression (2–6) as shown in supplementary Table S1.

### Statistical analysis

The significance of the association among PHB1 and LPLUNC1 expression in NPC was tested using the Spearman’s rank correlation coefficient. The differences between the groups were analysed using the Student’s *t*-test when there were only two groups or using one-way ANOVA when there were more than two groups. All statistical analyses were performed using the SPSS software (SPSS, Chicago, IL, USA). A two-tailed value of *P* < 0.05 was considered statistically significant (*P < 0.05, **P < 0.01, ***P < 0.001)

## Supplementary information

Supplymentary data

Supplymentary Table S1

Supplymentary Table S2
