# Supplementary material for: LPLUNC1 stabilises PHB1 by counteracting TRIM21-mediated ubiquitination to inhibit NF-κB activity in nasopharyngeal carcinoma
Source: Oncogene. 2019 Mar 18;38(25):5062–75. doi: 10.1038/s41388-019-0778-6 (PMC6756001; doi:10.1038/s41388-019-0778-6)
Supplement: Supplementary file 1 — Supplymentary data [file 41388_2019_778_MOESM1_ESM.pdf]

Figure S1

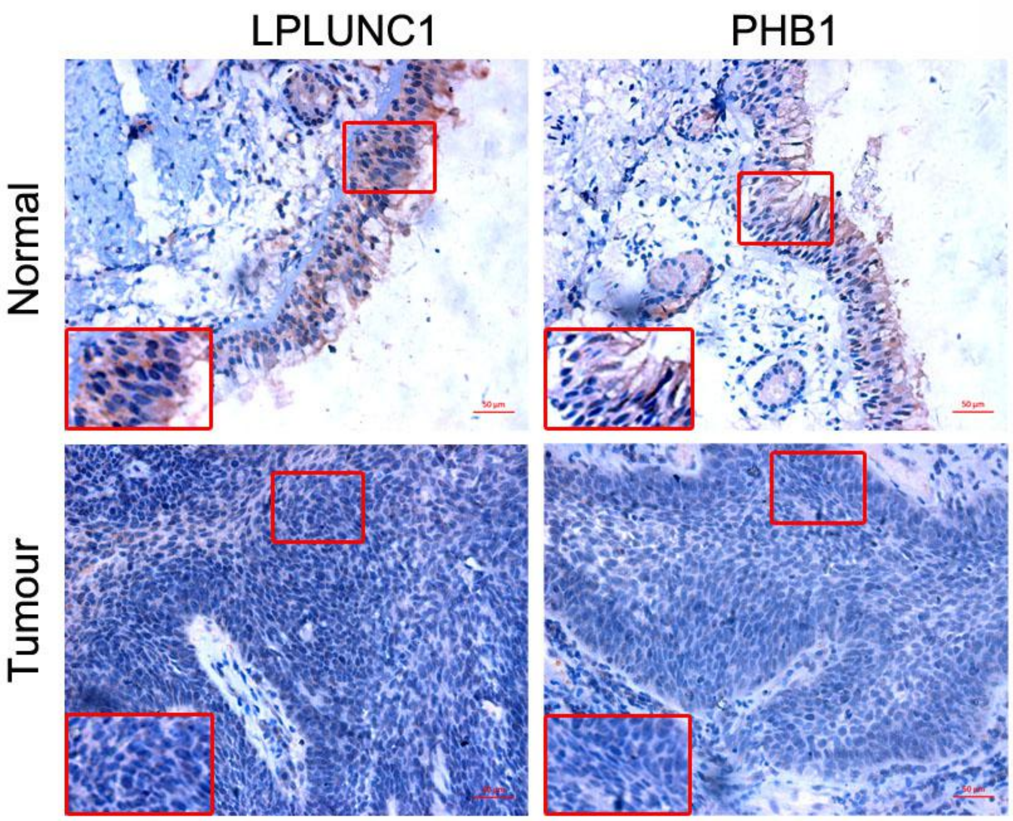

Figure S1. IHC analysis of LPLUNC1 and PHB1 expression in nasopharyngeal carcinoma (NPC) specimens and normal nasopharyngeal epithelium (NPE) specimens, Scale bar 50μm. Data shown are representative images.

Figure S2

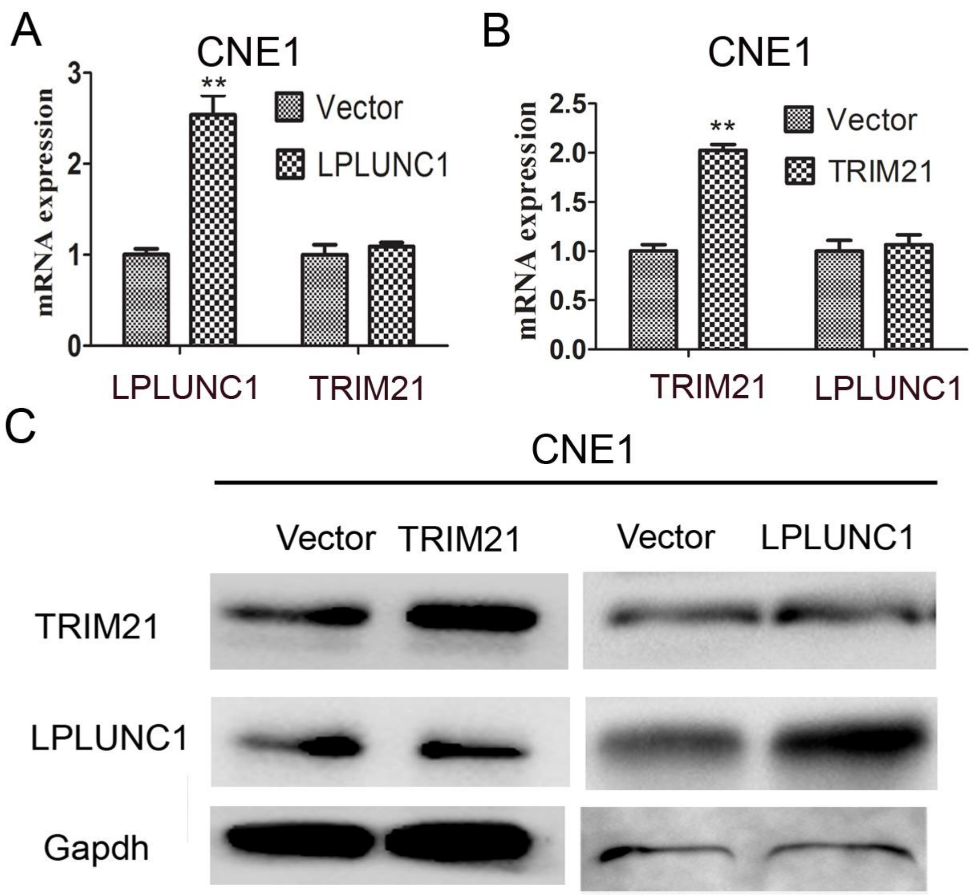

Figure S2 LPLUNC1 (A) and TRIM21 (B) mRNA level detected by real-time PCR in LPLUNC1 or TRIM21 overexpression CNE1 cells. (C) LPLUNC1 and TRIM21 protien level detected by western blotting in LPLUNC1 or TRIM21 overexpression CNE1 cells. Data shown are representative images or expressed as the mean ± s.d. of each group of cells from three separate experiments. (**P < 0.01 vs. vector, Student's t test).

Figure S3

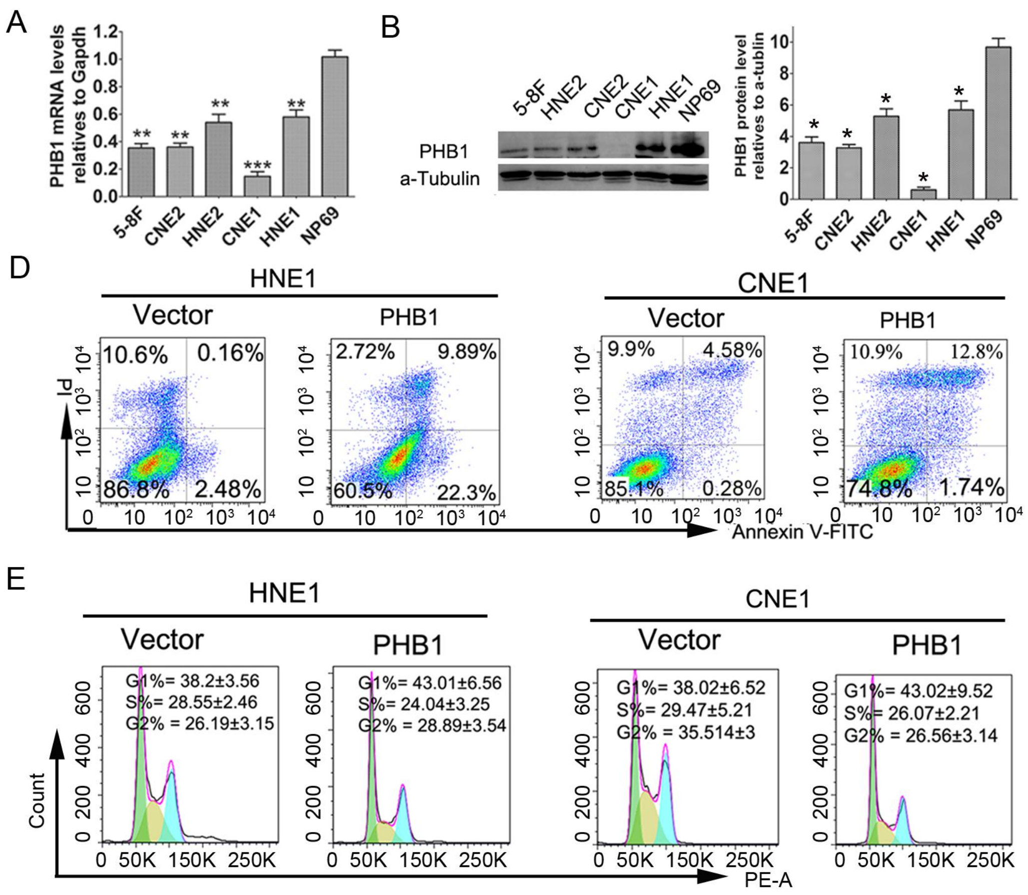

Figure S3. (A) PHB1 protein expression was analyzed by western blot. (B) Quantitative analysis of western blots with PHB1 expression in NPC cell lines. (C) Realtime PCR analysis of PHB1 mRNA expression in NPC cell lines compare with NP69 cells. (D) Images of Cell-cycle analysis by flow cytometry. (E) Images of Annexin V-FITC and PI double staining analysis of cell apoptosis by flow cytometry. Data shown are representative images or expressed as the mean ± s.d. of each group of cells from three separate experiments. (*P < 0.05; **P < 0.01 vs. the controls, Student's t-test).

Figure S4

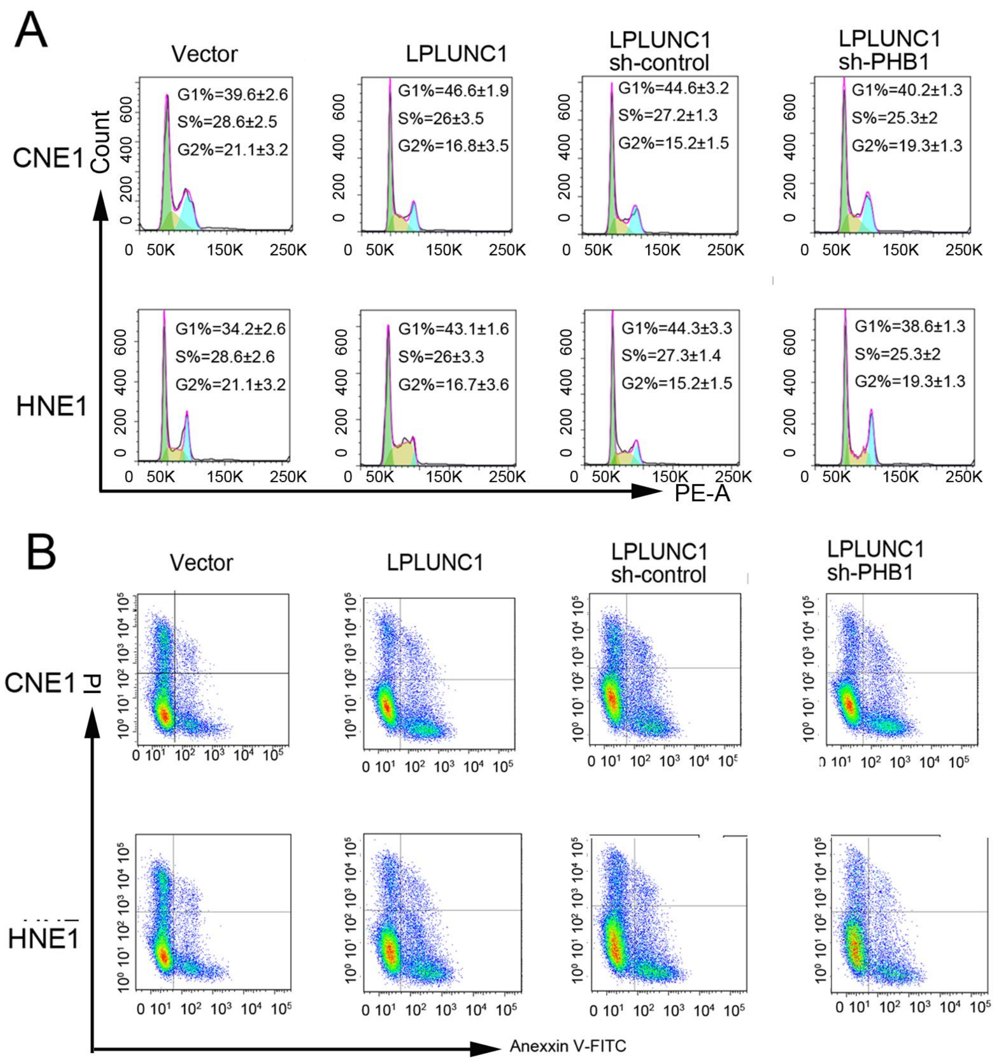

Figure S4 (A) Images of Cell-cycle analysis by flow cytometry. (B) Images of Annexin V-FITC and PI double staining analysis of cell apoptosis by flow cytometry in indicated groups. Data shown are representative images of each group of cells from three separate experiments.
