# Supplementary material for: LPLUNC1 stabilises PHB1 by counteracting TRIM21-mediated ubiquitination to inhibit NF-κB activity in nasopharyngeal carcinoma
Source: Oncogene. 2019 Mar 18;38(25):5062–75. doi: 10.1038/s41388-019-0778-6 (PMC6756001; doi:10.1038/s41388-019-0778-6)
Supplement: Supplementary file 2 — Supplymentary Table S1 [file 41388_2019_778_MOESM2_ESM.docx]

| **Table 1 Correlation between LPLUNC1 expression and PHB1 expression** | | | | | |
| --- | --- | --- | --- | --- | --- |
| **LPLUNC1** | **N** | **PHB1** | | **r** | **P** |
|  |  | **High expression** | **Low expression** |  |  |
| **High expression**  **Low expression** | 22  144 | 32  16  16 | 134  6  128 | 0.530 | 0.000 |
